# Challenges to and opportunities for the adoption and routine use of early warning indicators to monitor pediatric HIV drug resistance in Kenya

**DOI:** 10.1186/s12887-018-1209-5

**Published:** 2018-07-25

**Authors:** Nanlesta A. Pilgrim, Jerry Okal, James Matheka, Irene Mukui, Samuel Kalibala

**Affiliations:** 1 Population Council, 4301 Connecticut Avenue NW, Suite 280, Washington, DC 20008 USA; 2 Population Council, Nairobi, Kenya; 3 National AIDS & STI Control Programme, Nairobi, Kenya

## Abstract

**Background:**

Pediatric non-adherence to antiretroviral therapy (ART), loss to follow-up, and HIV drug resistance (HIVDR) are challenges to achieving UNAIDS’ targets of 90% of those diagnosed HIV-positive receiving treatment, and 90% of those receiving treatment achieving viral suppression. In Kenya, the pediatric population represents 8% of total HIV infections and pediatric virological failure is estimated at 33%. The monitoring of early warning indicators (EWIs) for HIVDR can help to identify and correct gaps in ART program functioning to improve HIV care and treatment outcomes. However, EWIs have not been integrated into health systems. We assessed challenges to the use of EWIs and solutions to challenges identified by frontline health administrators.

**Methods:**

We conducted key informant interviews with health administrators who were fully knowledgeable of the ART program at 23 pediatric ART sites in 18 counties across Kenya from May to June 2015. Thematic content analysis identified themes for three EWIs: on-time pill pick-up, retention in care, and virological suppression.

**Results:**

Nine themes—six at the facility level and three at the patient level—emerged as major challenges to EWI monitoring. At the facility level, themes centered on system issues (e.g., slow return of viral load results), staff shortages and inadequate adherence counseling skills, lack of effective patient tracking and linkage systems, and lack of support for health personnel. At the patient level, themes focused on stigma, non-disclosure of HIV status to children who are age eligible, and little engagement of guardians in the children’s care.

Practical solutions identified included the use of lay health workers (e.g., peer educators, community health workers) to implement a variety of care and treatment tasks, whole facility approaches to adherence counseling, adolescent peer support groups, and working with children directly as soon as they are age eligible.

**Discussion:**

The monitoring of EWIs has not been routine in health facilities in Kenya due to several challenges. However, facilities have implemented novel strategies to address some of these barriers. Future work is needed to assess whether scale-up of some of these approaches can aid in the effective use of EWIs and improving HIV care outcomes among the pediatric population.

## Background

In 2014, UNAIDS launched “90–90–90” targets aimed at ending the HIV epidemic whereby 90% of all people living with HIV are diagnosed, 90% of those diagnosed HIV-positive receive treatment, and 90% of those receiving treatment achieve viral suppression by 2020 [1]. However, there is need for more focused attention on achieving these targets among the pediatric population [2]. Globally, 2.6 million children younger than 15 years of age are living with HIV, 90% of whom reside in sub-Saharan Africa, and only 32% are accessing antiretroviral therapy (ART) [2]. In Kenya, children aged 0 to 14 accounted for 8% of total HIV infections (*n* = 120, 000) in 2016, with 45% in need of ART [3]. With such rates, achieving the second and third “90s” among the pediatric population is in danger. It is important that these targets are achieved given that the pediatric population faces lifelong treatment and there are limited treatment options available [2]. The prevention of HIV drug resistance (HIVDR) is therefore critical within the pediatric population.

Existing research finds that the pediatric population living with HIV are at high risk of virological failure of ART and acquiring drug-resistant HIV, with some studies placing drug resistance estimates as high as 60–90% [2, 4, 5]. A 2013 study among 100 Kenyan children, aged 18 months to 12 years, reported 34% of them experienced virological failure and 68% of those with failure had drug-resistant mutations [6]. Similarly, a 2014 Kenyan study of 462 children younger than 5 years in 15 sentinel sites reported 33% of children experienced virological failure with a higher drug resistant mutation rate of 88% [7]. Poor adherence, which is prevalent during early childhood and adolescence, is a significant contributor to failure [8, 9]. One review reported wide adherence estimates, ranging from 49 to 100% among pediatric populations in low and middle income countries [10]. Moreover, loss to follow-up remains a key concern. A recent systematic literature review found that one year retention rates ranged from 71 to 95% among 31,877 African children with 73% of those who were not retained being due to loss to follow-up, and 27% were confirmed to have died [2, 11].

Given the high rates of virologic failure and drug-resistant HIV as well as widely variable rates of adherence and loss to follow-up, there is a need to strengthen health systems to support retention in care and ART adherence among the pediatric population if the ambitious UNAIDS targets are to be realized. The monitoring of early warning indicators (EWIs), developed by the World Health Organization (WHO) in 2004 and refined in 2011, can help to identify and correct gaps in ART program functioning and quality of service delivery to aid in the prevention of HIVDR, improve patient retention in care, and increase adherence [8, 12]. The five EWIs monitor factors that are associated with HIVDR related to patient care, patient behavior, and clinic management (Table 1). If implemented and monitored consistently, EWIs can provide an evidence base for programmatic change and/or public health action to prevent and address HIVDR or virologic failure among the pediatric population [12].

**Table 1:** Early warning indicators for HIV drug resistance

On-time pill pick-up: % of patients with 100% on-time drug pick-up during the first 12 months of ART or during a specified time period Retention in care: % of patients retained in care 12 months after ART initiation Drug stockout: % of months with any day(s) of stock out of any routinely dispensed ARV drug Prescribing practices: % of ART prescriptions congruent with national/international guidelines Viral load suppression: % of patients with viral load < 1000 copies/mL 12 months after ART initiation	

The positive outcomes that can be realized through the monitoring of the EWIs are dependent on their uptake by clinic and program management as well as their regular use in deciding how to improve program functioning and quality of service delivery. In 2012, the Kenyan National AIDS & STI Control Programme (NASCOP) assessed the use of EWIs in 32 of the approximately 1032 pediatric ART sites across Kenya and found the sites had good prescribing practice (98%) but moderate to poor patient retention in care (69% of patients retained at 12 months), retention on first line therapy (50%), and appointment keeping (29% kept > 80% of appointments) [13]. Results also showed that the routine utilization of EWIs within health facilities was a challenge and their use has not been introduced across the country.

With the need to expand the use of EWIs as a method for reducing HIVDR as well as improving adherence and patient retention in care, the current study was conducted with frontline health administrators to assess the challenges to routine utilization of EWIs and to identify strategies to increase the uptake and utilization of EWIs within pediatric facilities.

## Methods

### Sample

Key informant interviews (KIIs) were conducted in 23 pediatric ART facilities, between May and June 2015, with the facility official who was fully knowledgeable of the pediatric ART program and procedures. The identified individuals were typically the Officer in Charge or a pediatric provider. The pediatric sites were a subset of the 32 sites, where the 2012 EWI monitoring assessments were conducted by NASCOP [13]. Stratified random sampling by geographic region, facility type (e.g., health center) and administration (e.g., Ministry of Health [MoH], faith-based organization [FBO]) were used to select the facilities. Facilities located in the former North Eastern province of Kenya were excluded due to political unrest at the time of the data collection. The facilities included represent 18 of the 47 counties and 7 of the 8 former provinces of Kenya.

### Recruitment and interview procedures

Prior to KIIs, the investigators called managers at each facility to explain the purpose of the study and request the support of the Officer in Charge in identifying the most knowledgeable individual to take part in KIIs. A signed letter requesting their support from NASCOP and the MoH was also provided. An appointment was then scheduled for the completion of the KIIs. KIIs were completed with the Officer in Charge of the health facilities. The Officers in Charge were nurses, clinical officers, or doctors.

The KIIs were conducted in English in a private location at each facility and lasted approximately 60 minutes. Trained research assistants with clinical backgrounds conducted all KIIs. Research assistants used a semi-structured interview guide to facilitate the discussion. The KII guide consisted of questions that generated discussion on facility pediatric ART treatment procedures, existing EWI monitoring procedures, and identification of strategies to improve EWI monitoring (Table 2).

**Table 2:** Interview questions asked of health facility officers in charge

1. Please describe the methods this facility uses to monitor HIV drug resistance for the pediatric population. a. What challenges, if any, have you experienced using these methods to monitor drug resistance? b. What are the positive aspects of using these methods to monitor drug resistance? 2. How effective has any of these drug resistance monitoring systems been in identifying possible drug resistance in the pediatric population? 3. Please describe any standards and procedures regarding conducting pill counts with pediatric ART patients at this facility. a. What challenges or barriers does this facility experience regarding conducting pill counts with pediatric ART patients? b. How can these barriers or challenges be addressed? 4. Please describe any standards and procedures regarding the conduct of adherence counseling with pediatric ART patients at this facility. a. What challenges or barriers does this facility experience regarding conducting adherence counseling with pediatric ART patients? b. How can these barriers or challenges be addressed? 5. Please describe any standards and procedures for tracking or tracing pediatric ART patients who miss appointments and drug pickups at this facility. a. What challenges or barriers does this facility experience regarding tracking or tracing ART patients? What about among the pediatric ART patients? b. How can these barriers or challenges be addressed? 6. Does this facility have the equipment and qualified staff to conduct viral load testing? If yes, a. Please describe any challenges or barriers to conducting routine viral load testing at this facility. b. In your opinion, how can these barriers or challenges be addressed? c. What works best in conducting routing viral load testing? If no, a. Please describe the procedures regarding viral load testing with pediatric patients? b. What challenges or barriers does this facility encounter with viral load testing? c. In your opinion, how can these barriers or challenges be addressed? d. What works best in conducting routing viral load testing? 7. Please describe how the current facility practices regarding pill counts, adherence counseling, and/or patient tracing may affect the quality of records needed for pediatric ART monitoring at this facility. 8. Overall, are the ART medical and pharmaceutical records at this facility well-maintained, or are there some gaps in recording the necessary information? a. Please describe any factors or challenges to maintaining complete and up-to-date ART records. 9. What interventions would you recommend to improve routine EWI monitoring at your facility?	

### Analyses

All KIIs were audio recorded and transcribed verbatim for analysis. Trained research team members verified all transcripts against the original audiotapes to ensure that the transcriptions were accurate. Thematic content analysis, a research method for the subjective interpretation of the content of text data through the systematic classification process of identifying themes or patterns, was used [14]. Themes identifying key factors influencing the routine use and monitoring of EWIs, the challenges and opportunities for EWI monitoring, and strategies facilities have used to overcome challenges were identified by research staff (NP, JO, JM). Identification of themes were an iterative process whereby themes were redefined or merged based on emerging patterns in the data [15]. JO and JM initiated the process by reading and open coding all transcripts and noting all topics raised by the respondents. JO next consolidated topics into major themes, whereby some topics were expanded upon while others were eliminated or merged. Throughout the analytic process, NP reviewed all themes derived from the analyses. The differences in themes by type of clinics were minimal and therefore, we focus on crosscutting findings. Discussions around dispensing practices and pharmacy stock-outs were limited and therefore, the results focus on factors influencing on-time medication pick-up, retention on ART and care, and virological suppression.

### Ethical approval

This protocol was reviewed and approved by the Population Council Institutional Review Board and the Kenyatta National Hospital/University of Nairobi Ethics & Research Committee. To protect facility Officers in Charge, we did not collect any personal identifying information to ensure that they could not be identified. Facility Officers in Charge provided verbal consent before being interviewed.

## Results

KIIs were conducted with participants from five types of facilities: teaching/referral hospital (*n* = 2), provincial hospital (*n* = 8), district hospital (*n* = 6), sub-district hospitals (*n* = 3), and health center/dispensary (*n* = 4). Seventeen facilities were managed by the county government, three by the MoH, and three by FBOs. None of the facilities were currently using EWIs. Table 3 presents the nine themes that emerged across the three EWIs that yielded the most discussion - on-time pill pick-up, retention in care, and virological suppression - and the proportion of transcripts with the theme.

**Table 3:** Themes and % of transcripts with theme organized by EWI

Theme	**%**
On-time pill pick-up	
*Facility level*	
Inappropriate forms to record pediatric information.	39.1
Variable use of pill count to assess adherence.	34.7
Staff shortages and inadequate adherence counseling skills	47.8
*Patient level*	
Non-disclosure of HIV status to the child hinders adherence	69.6
Stigma hinders adherence	30.4
Retention in care	
*Facility level*	
Lay providers require support	82.6
A need for a national tracking system and tracking policies	21.7
*Patient level*	
Guardians pose a challenge to pediatric retention in care	52.2
Viral load suppression	
*Facility level*	
Systemic issues prohibited viral load measurement	95.7

### On-time pill pick-up

Five themes emerged that influenced on-time pill pick-up. At the facility level, low human resource capacity and inadequate adherence counseling skills; variable or non-usage of pill count to assess adherence and inappropriate clinical forms to record pediatric information affected providers ability to track medication use. At the patient level, non-disclosure of HIV status to children and stigma hindered adherence to ART and therefore, negatively affected medication pick-up. Within each theme, any associated strategies respondents have used to address the challenges encountered are presented.

### Facility level

#### Inappropriate forms to record pediatric information

Participants explained that there was a lack of space on standard clinical forms to record dosage information and other important notes regarding monitoring such as pill counts and adherence counseling. This critical information impeded patient care because there was no way to appropriately and efficiently keep track of pediatric information. While some providers added the information using an extra piece of paper, the process is not standardized and therefore, the next provider seeing the patient might not fill out the information.

The spaces provided are not adequate. For example, on the space of the drug that I am prescribing for the client, there is no space to prescribe the dosage. It’s only the type of drug but the dosage is not there…. [I] wish that it had enough adequate space for us to include the drug dosage. (Provincial hospital, MoH managed)

For the pediatric population I thought we would have an extra blue card, a different one designed for them because some of the information here is not meant for the pediatrics. (Provincial hospital, County government managed)

#### Variable use of pill count to assess adherence

Pill count procedures varied across facilities, with some respondents reporting conducting pill counts every visit, some relying on guardians’ reports, and others not conducting pill counts at all. Respondents questioned the usefulness of pill counts, especially since the clinician forms did not have a space to record the information. Moreover, they explained that since most pediatric drugs were in liquid formula, it was difficult to get a correct estimate of the remaining drugs if the guardians forgot to bring the bottles. Other times, they could not engage with the pediatric clients themselves because clinic hours occurred during school times. Therefore, they were unable to assess drug usage.

We don’t have anywhere to record those pill counts, we haven’t put measures on how to put pill counts on records. (Health center, County government managed)

Our main challenge as I had told you earlier is most of the population, especially from 5-14 [years old]…is still schooling…. That time for schooling, you only see the caretaker coming or the treatment supporter coming to collect the drugs for the child, while this child was supposed to visit. Yeah, so mainly the challenge we are getting especially where the clients are concerned the failure to visit the clinic in time. (District hospital, FBO managed)

#### Staff shortages and inadequate adherence counseling skills

Participants recounted a number of factors at their facility that negatively impacted adherence counseling, on-time medication pick-up, and retention in care (EWI 2). Staff shortages resulted in patients receiving shortened and at times, no counseling, due to competing demands among the providers and the increasing volumes of patients. They also noted that providers needed more training to provide specialized counseling and psychosocial support services to their clients. Additionally, high patient volumes resulted in incomplete patient records. While facility staff endeavored at the end of the day to complete all records, they were often overwhelmed, and records remained incomplete. Even with some facilities having electronic medical records (EMR), many only had 1–2 computers. When coupled with unpredictable electricity, they relied on paper-based record keeping systems before entry into the EMR.

Our facility workload is very large, even though we need more time to counsel, sometimes we shorten our counseling period because we have other patients who are waiting to be seen…. So at least when we deal with the staffing issues we will have dealt with the challenge. (Health center, County government managed)

We make sure that everything is documented by the end of the day, but sometimes, the workload is too much for us, we find that we have so much to do at the end of the day…. We need more staff, record officers, we are doing work which is not ours, it’s for records, filling the files, tracking the clients. (District hospital, County government managed)

Though all participants called for the deployment of more health staff to cope with the high number of clients seeking services, some respondents described strategies they have instituted to combat the challenges faced. One strategy has been to train peer educators, community health workers, and people living with HIV to help with adherence counseling of both adult and pediatric populations. In fact, peer educators also assist with pill count and tracing of clients who miss appointments.

Okay, the peer educators can show you the record where they capture the adherence counseling and also the patient’s file has everything. In the file there is a form for adherence counseling. (Health center, County government managed)

Respondent: The counseling is done by trained personnel on adherence counseling. We also have PLP taking the clients through adherence counseling.

Moderator: What is PLP?

Respondent: That is people living positive.

Moderator: Okay, they also do the counseling for…

Respondent: Adherence because we have trained them. (Provincial hospital, MoH managed)

Another strategy has been to take a whole facility approach to adherence counseling. That is, everyone that a client encounters at the facility—from front desk staff to pharmacist to peer educator—has been trained on adherence counseling so that consistent adherence messaging is provided to all clients. While facilities were short-staffed, they endeavored for adherence messaging to be delivered at each point of care. Similarly, a few facilities described regularly (e.g., monthly) bringing together different departments to discuss any clients who might be heading toward drug resistance and implementing steps to address the problem.

The main adherence counseling is done by the nurse, because we require a professional to do the enrolment as we empower the client with adequate information on care and treatment and everywhere else adherence continues because the clinician will talk about it, the peer educator will talk about it, the records person will talk about it, the pharmacist, the nutritionist the same and the like, it’s for each…. Adherence counseling is done on every visit and we reinforce it especially where we identify a gap. (Provincial hospital, County government managed)

To address the inefficient record keeping system, at least one facility hired a records officer dedicated solely to ensuring that all records were kept updated and complete.

We have our records office being managed by our qualified health information records officer. She has all the registers with her, the daily activity register. She is the one who manages the diary, she manages the ART register and after every activity, she sits down to go through the day’s work, identify where the gaps are and they compare their results with the peer educators who have also been asked to monitor all the clients booked for the day’s work. Then they bring their data together to see whether there is any data remaining so the records are well kept in the records office. (Provincial hospital, County government managed)

### Patient level

#### Non-disclosure of HIV status to the child hinders adherence

Among the pediatric population, discussions around medication adherence and drug resistance are usually held with guardians, who may or may not be the child’s parents. Ideally, participants prefer to start adherence counseling with the pediatric patient as early as possible so that the child understands the importance of taking medication on-time and staying in care. However, many guardians remain reluctant to disclose to their children that they are living with HIV. In return, some children saw no reason to take the medication and stopped, thus affecting on-time pick-up of medications.

So I think pediatrics is a challenge on adherence. Then the other problem with the pediatrics is disclosure because you question why: “Why am I taking? What are these for?” Most too often than not they won’t tell them they are taking drugs for HIV. Like the caregivers, they won’t tell them the truth that they are taking them for the HIV disease so they would take and take and sometimes they get tired of taking and say “I won’t take again...” till you explain to them why they are taking. We have even had teenagers taking ARVs and don’t know they are taking ARVs. (Provincial hospital, County government managed)

Participants explained that they engage in regular adherence counseling with guardians, where a key component is emphasizing early disclosure so that the child is prepared well in advance to transition to adult care. When they have succeeded, they engage the child as early as possible (for some facilities as young as age six) in their care focusing on understanding HIV, the importance of medication adherence, and the importance of keeping appointments. Some participants noted times when children come to the clinic without their parents because of the counseling the child received.

The issues, especially if they are not disclosed, parents have not disclosed, so it’s a problem, they refuse to come back. They are as if they don’t want to take the drugs because the parents have not explained to them why they are taking drugs. They say why are they taking drugs and others are not taking. So we get them into groups and explain to them why they are taking drugs and we involve their parents, that is why we are able to retain them in here. (District hospital, County government managed)

When the child is ten years, we like including them as early as possible. So they are able to understand. Ten years I am imagining it’s a child in class four, so this is a child who is able to understand. So we help them understand the importance of taking the medicine and we assist them in knowing how many they are supposed to take and we involve them in the counting so that they can appreciate how they need to take their drugs and what I expect the next time they come over. (Provincial hospital, County government managed)

#### Stigma hinders adherence

Participants described that experience of stigma, especially in the school settings, negatively impacted adherence to medications among the pediatric population, especially those in adolescence. Some school-going adolescents live in dormitories and when their status is known, they might be ridiculed or shunned. In response, they would take their medications intermittently, such as when they return home. By the time they see their clinician, they could have developed drug resistance.

Our adolescents, they experience a lot of challenges when they go to school…. The environment at school may be hostile and he will abandon treatment. How to access the dormitory is a problem. How to take their medication because…it may be during class time is a problem. So you find that they keep the drug until they feel they are free, that is when they take the drugs. So it has led to drug resistance in children. (Provincial hospital, County government managed)

In an attempt to counteract the stigma encountered, some facilities separated clinic days for younger and older pediatric clients, recognizing that each group has their own special needs. Specifically, for older pediatric patients, some facilities formed pediatric support groups.

For the pediatric patients, we also have some groups, pediatric support group. We have children support groups, when we also follow them and talk to them, so that they can be able to interact together with those who are positive and those who are not. (District hospital, County government managed)

### Retention in care

Three themes emerged that influenced retention in care. At the facility level, lack of necessary support for lay health workers and lack of a tracking system and policies, negatively affected the ability to retain the pediatric population in care. At the patient level, challenges with pediatric guardians were the predominant barrier to retention. Within each theme, any associated strategies respondents have used to address the challenges encountered are presented.

### Facility level

#### Lay health workers require support

To facilitate patient retention, participants described relying heavily on lay health workers (e.g., peer educators, volunteers, and community health workers) to conduct tracing of patients who miss appointments. Peer educators initiated outreach via mobile phones and short message services (SMS) to guardians and patients (if old enough) to reschedule missed appointments. If the patient does not have a mobile phone or cannot be reached, their information is given to community health workers to trace them within the community. If the tracing is successful, the clients are brought back to care and intensive counseling is initiated to understand the reasons for missing the appointment and to prevent loss to follow up. If unsuccessful, some facilities mark them as lost to follow-up while others wait until they reappear.

The volunteer who works here is conversant with most of the clients that come from the area that she comes from…. Or she is able to know somebody who comes from an area that is nearer one of the clients so we are able to track them that way. (Sub-district hospital, Country government managed)

If they don’t come, we call volunteers or the community health worker to follow them. We also have the SMS system, we send them an SMS daily. (District hospital, County government managed)

However, some participants explained that the ability to trace patients has been hindered by a lack of financial resources to support lay health workers. For example, funds do not exist to purchase airtime to make calls or send SMS to clients nor are there funds for transportation to physically trace clients in the community. Participants describe instances where staff have used their own money to buy airtime to make calls or send SMS. However, staff and volunteers have become increasingly reluctant to use their own money due to the high volume and expense. As a result, little to no effort is made to retain clients in care when they do not show up for appointments.

It all amounts to financial support. For the follow up, we will need financial support. One, they need airtime. Two, in terms of motorcycles or vehicles, they will need fuel. (Teaching/referral hospital, MoH managed)

Some of those patients don’t have phone numbers and there is no money provided for physical tracing. So, when they don’t have a phone and don’t come, we just wait for them. We don’t trace them physically. (Sub-district hospital, County government managed)

#### A need for a national tracking system and tracking policies

Closely linked to the ability to trace clients is the need for tracking policies and a national tracking system. A few respondents noted that guidelines on how to track clients did not exist. For staff safety, guidelines should be created and distributed to facilities.

How do I do a follow up? How am I covered with the policy, in case anything happens to me there? Is this policy designed in a way to protect me? …you could go somewhere you find [gangs], you find them armed with knives, so is there anything to show if I go there and anything happens? So the guidelines [would] really assist, …the guidelines should come officially in this manner so that you can just put it there… even when clients come you can point out to the client and say, you see what the government says in this and this. (Health center, Country government managed)

For both pediatric and adult populations, participants expressed frustration over patients moving between health care facilities and not being able to adequately track them or record the information within their records. That is, if a patient moves away for a short while, they might register at a different clinic and receive medications from that facility. When they return, they come back to their original health facility. While some providers call the other facilities to fill in the necessary information to have complete records, there is no standardized process of doing this. Additionally, they must rely on patient self-report that they were under the care of another provider when they were absent from the facility.

Clients on transit are a challenge and those are the things we experience as a facility, if NASCOP had a mechanism like a national ID card such that all clients who are enrolled to care and treatment are able to be tracked at one point, it will help us. (Provincial hospital, County government managed)

### Patient level

#### Guardians pose a challenge to pediatric retention in care

Participants explained that retention in care for their pediatric populations is a major problem primarily due to challenges with caregivers. In addition to the non-disclosure previously noted, participants explained that some caregivers had little or no interest in being engaged in their children’s/ward’s health care and therefore, neither brought the child to their appointment nor made sure they took their medications. Some children, especially those who are orphans, switched caregivers frequently with the new caregivers often unaware of the child’s HIV status. Therefore, the continuity of the child’s care is compromised.

Getting the relative’s contact becomes hard because whoever has been the treatment support sometimes when you call back they say they do not know the child, or the child went with other relatives. They do not know how the child is fairing on, so it becomes hard because they hand over from one person to another. (Provincial hospital, County government managed)

The father is there but he is not cooperative because when I asked the child to be accompanied by him, he doesn’t come. I have never seen him…. The other relatives are not near. He only stays with the father and the mother is not there. She passed on. (District hospital, FBO managed)

In light of the challenges posed by guardians, some facilities instituted practices to help increase retention among the pediatric population. These practices included a community approach to pediatric care, whereby providers identify multiple individuals within the child’s social circle, including relatives and teachers, who can support the child in their care and treatment. They also collected multiple forms of contact information from the child’s current guardians of all relatives the child could potentially live with. As stated previously, where possible, they engage the children early so they understand the importance of visiting the facility regularly.

For the pediatric, we try to have several phone numbers on how we can reach them. If we can have two or three caregivers who stay with the child, if at all we are not able to reach one, we can try the other one. (Sub-district hospital, County government managed)

Maybe community—identifying other people who can be able to support the child outside that person who comes with the child. Addressing the family as a whole so that when one person is not there, the others can be able to sit in for the main one, and also involving the child quite early and making the child understand the importance of drug adherence. (Provincial hospital, County government managed)

### Viral load suppression

One theme at the facility level emerged as a challenge to monitoring potential virological failure.

#### Systemic issues prohibited viral load measurement

Participants stated that they relied mainly on CD4 counts and clinical staging of patients to aid in the assessment of drug resistance as there were several systemic issues that prohibited the measurement of viral loads. Although all participants noted they had access to viral load testing, either by having viral load machines on site or sending specimens to a neighboring facility, many identified several issues prohibiting the on-time measurement of viral loads: constant stock-out of dry blood spot filter paper and reagents, machine breakdown, long turnaround time (e.g., 2–3 months) to receive testing results, rejection of samples due to poor packaging, and samples getting spoiled during transportation. Some facilities were located far from a testing site and lacked adequate transport, making it difficult to transport specimen in a timely manner.

CD4 we did not have that much of the challenge. The turnaround time was short, we would get the results even in a week’s time. But for viral load the turnaround time is very long. The thing is that by the time I bleed until I get my results, even 2/3 months can go by. So that is not appropriate because you need to have results as soon as possible so that we can make decisions as soon as we wish to. (Provincial hospital, County government managed)

Participants emphasized the need for timely replenishment of the necessary supplies to conduct appropriate tests because the current system negatively impacts the quality of care provided to patients and the degree to which patients engage in their care. Participants explained that patients stop coming for care after being repeatedly informed that the facility did not have the appropriate supplies to test them or have not yet received their test results. Thus, it is closely linked to retention in care.

Provide a viral load machine…and also have continuous supply of filter papers or what is required for the viral loads to be done so that we can be able to meet our targets. (Provincial hospital, MoH managed)

Yes, erratic supply also demotivates the client actually. You come today and you are told it’s not there; you come next time you are told it’s not there, so you will not bother again and just forget about it. (Provincial hospital, MoH managed)

## Discussion

In this study, we conducted KIIs with frontline heads of facilities to assess the challenges to routine utilization of the EWIs and to identify strategies to increase the uptake and utilization of EWIs within pediatric facilities. We identified challenges at the facility level as well as the patient level associated with the monitoring of EWIs.

For EWI monitoring to be used routinely in the provision of care and treatment to pediatric patients, there is a need to address staff shortage. In our study, some facilities filled the gap by using lay health workers to provide several services, including adherence counseling, pill counting, and client tracking. Task shifting and sharing within the health system has been a key HIV care and treatment implementation strategy and the use of lay health workers can aid in ensuring high quality care is provided [16]. For EWI to become more routinized, capitalizing on the strengths of lay health workers would contribute to alleviating the concerns of overburdened and short-staffed health system. However, they will require the necessary resources, support, and training to be effective. For example, if trained appropriately, a dedicated lay health worker at each facility can be used to regularly abstract the information from clinical records to calculate the EWIs. Additionally, the rapid initiation of EMR systems in facilities can facilitate the process. This can allow for timely retrieval of EWI results and the implementation of steps to address barriers hindering optimal performance. Additionally, there is need to conduct studies to project how the use of lay health workers can aid the health system on a larger scale.

The monitoring of EWIs is insufficient without equipping health professionals with the necessary skills to combat the barriers linked to on-time medication pick-up or retention in care. Training in the provision of psychosocial support, especially adherence counseling, is urgently needed. In a few facilities, a paradigm shift to training has occurred whereby everyone the patient encounters during their visit has received adherence counseling training, and this could be implemented on a larger scale. This paradigm of operation serves to reinforce positive messages at all levels. It also ensures that the patient receives the messaging even when the clinician does not have the time to provide counseling. Further study is needed to assess whether this approach is linked to increased adherence and retention in care.

There is also a need to establish and expand psychosocial and peer support groups for both pediatric patients and their caregivers. Early childhood and adolescence is a time of opportunity but it is also a time when children begin to form their identities [17]. As such, they are particularly susceptible to stigma, which can play a detrimental role in their physical, mental and sexual health and development [18, 19]. Therefore, it is critical that the necessary youth-sensitive, age-specific psychosocial and peer support groups are available for this population at health care facilities or within their communities. Peer support groups have been shown to be successful in improving adolescents’ emotional wellbeing and positively influencing medical outcomes, including medication adherence [20, 21]. These types of groups are also needed for caregivers, who are often reluctant to disclose to their children their HIV status or who are not as engaged in their children’s care and treatment. This type of reaction by caregivers is often driven by stigma and discrimination, whereby they try to preserve their children’s ‘normal’ childhood by protecting them from the potential stigma or discrimination they might encounter as a result of being known to be living with HIV [22, 23]. However, the WHO recommends that children of school age, six years and above, should be told their HIV status and younger children be told their status incrementally in preparation for full disclosure because there is evidence of health benefits and little evidence of psychological or emotional harm from disclosure of HIV status to HIV-positive children [24]. As such, there is a need for guardians to receive ongoing support as they prepare children for the adjustment process of living with HIV, addressing the associated life challenges, and becoming self-sufficient and independent [22–24]. Standardized protocols are also needed across facilities to help track children receiving HIV care. These could include the provision of forms to allow for the collection of multiple options of contact information for different potential caregivers of children.

Investments are needed to develop and improve facility systems to make the routine monitoring and use of EWIs a reality. The use of mobile technologies to support the achievement of health objectives has the potential to transform health service delivery, including health promotion, information access, health awareness raising, and decision support systems as well as enable behavior change and improve health outcomes in resource-limited settings [25–28]. For example, two randomized controlled trials in Kenya demonstrated improvements in ART adherence using mobile health platforms [26, 27]. Capitalizing on the proliferation of mobile technology across sub-Saharan Africa provides opportunities for improving EWIs. It can also be used in the design and implementation of a referral and linkage system to reduce loss to follow-up and ensure continuity of care among the pediatric population [29]. For example, one pilot study in Kenya utilized both internet-based coordination and text messaging to address barriers and improve the provision of early infant diagnosis of HIV [29]. The procurement system for medical supplies should also be regularly evaluated to prevent the stock-out of necessary supplies.

### Limitations

This study had limitations. The data for this study were self-reported. As such, the data generated should be assessed being mindful of the likely impact of social desirability bias, comprehension, and limitations of recall accuracy. However, qualitative interviews provided detailed insights into the key challenges faced by facilities in monitoring and using EWIs and possible strategies that can be expanded to facilitate their regular use. Only one person per facility was interviewed which increases the risk of bias but the interviewee was the Officer in Charge, whose responsibilities included having a wide breath of knowledge of the facilities processes. Thematic content analysis uses subjective interpretation of data. However, the credibility of the themes derived was checked through an external process whereby a data interpretation meeting was conducted with key stakeholders in Kenya, including health care providers [30]. The results were presented, and the key stakeholders confirmed the accuracy of the themes. We did not directly interview family caregivers of the children and might have missed other salient issues concerning challenges to retention and on time pill pick up. The paper does not present results on dispensing practices and pharmacy stock-outs as limited discussions emerged on these two EWIs.

## Conclusion

The routine monitoring and use of EWIs has the potential to significantly contribute toward achieving the UNAIDs’ targets of 90% retention on ART with 90% viral suppression rates on first-line therapy. The usefulness of EWIs is negatively impacted by weaknesses within the health system (e.g., staff shortages, long turnaround times for viral load results, lack of filter paper) as well as patient-level factors (e.g., guardian challenges and stigma). However, facilities have implemented strategies (e.g., use of lay health workers) to address some of these barriers. Future work is needed to assess whether scale-up of some of these approaches can aid in the effective use of EWIs as well as improving HIV care outcomes among the pediatric population.

## Abbreviations

ART: Antiretoviral therapy
EMR: Electronic medical records
EWI: Early warning indicators
FBO: Faith-based organization
HIV: Human immunodeficiency virus
HIVDR: HIV drug resistance
KII: Key informant interviews
MOH: Ministry of Health
NASCOP: Kenyan National AIDS & STI Control Programme
SMS: Short message services
UNAIDS: Joint United Nations Programme on HIV/AIDS
